# Grandparents, family solidarity and the division of housework: evidence from the Italian case

**DOI:** 10.1186/s41118-022-00168-4

**Published:** 2022-06-15

**Authors:** Marco Albertini, Marco Tosi

**Affiliations:** 1 Department of Political and Social Sciences, University of Bologna, Bologna, Italy; 2 Department of Statistical Sciences, University of Padua, Padua, Italy

**Keywords:** Grandparents, Gender, Housework, Household labour, Child care

## Abstract

As a consequence of recent socio-demographic trends and labour market transformations the role of grandparental support has become pivotal in individuals’ and households’ life courses. In Southern European countries the availability of grandparents affects young couples’ labour market participation and fertility decisions. In the present paper, it is asked if the potential availability of social support from the older family generation is associated with more or less inequality in the division of unpaid housework in couples with minor children, in Italy. Using data from the 2016 Family and Social Subjects survey it is shown that while there is not a clear relation between intergenerational face-to-face contacts and the symmetry of the division of household labour, adult children and older (grand)parents coresidence is associated with a more gender-equal sharing of housework within couples, arguably because co-residing grandparents take on the execution of a number of household tasks. The observed effect is comparable to that of hiring a paid housekeeper and higher than hiring a babysitter. Thus, despite one may think that three-generation households are characterized by a culture of traditional norms, our findings indicate that they have a more gender-equal division of housework.

## Introduction

In the last decades, macro-socio-demographic trends have contributed to making the role of grandparents increasingly salient in European societies, and even more so in Southern European ones. On the one hand, the growing percentage of three- and four-generation families and the improvement of heathy life conditions have made increasingly common to spend longer periods of life with active grandparents (Leopold & Skopek, 2015; Margolis & Wright, 2017). On the other hand, it has become very frequent that children grow in families where both parents work full-time and, due to insufficient availability of childcare services, they are regularly taken care by their grandparents, often on a daily basis (Albertini, 2016; Hank & Buber, 2009). The support provided by grandparents in upbringing minor children is relevant in many aspects of parents’ lives, including labour market participation and fertility decisions (Aassve et al., 2012; Thomese & Liefbroer, 2013).

Within this social context, it is important to investigate how the presence of grandparents and the support they provide may affect the lives of young Italian couples. In the present paper, we investigate the association between the intensity of face-to-face contacts between older (grand)parents and their adult children—which we conceptualize as a proxy of potential/actual availability of informal support provision—and an important aspect of heterosexual couples’ life: the asymmetry in the distribution of household work. Couples’ division of housework may depend on the presence of grandparents who are usually able to alleviate the burden of time-consuming household tasks. Indeed, Italian grandparents tend to be younger, healthier and more socially involved than their European counterparts (Pasqualini et al., 2021; Zamberletti et al., 2018).

Using data from the 2016 Family and Social Subjects survey, we analyse grandparent–grandchildren contact frequency as an important part of intergenerational family solidarity, namely associational solidarity (Bengtson & Roberts, 1991). Face-to-face interaction is the most basic way in which family members share experiences and exchange practical and instrumental support. Frequent meetings are restricted by geographical distance (structural solidarity) and residential choices including the one of living in the same household that is often regarded as a form of support that family generations provide to each other, particularly in Southern Europe and among the lower social classes (Albertini, 2008; Albertini & Kohli, 2013; Glaser et al., 2018; Margolis & Wright, 2017).

## The role of grandparents in Italian families

Numerous studies have shown how grandparent’s emotional, financial and time investment in their grandchildren has acquired a pivotal role in the life course of different family generations within European households (e.g. Glaser & Hank, 2018). What recent analyses clearly show is that the provision of grandparental help is ubiquitous across the different European countries but, at the same time, the intensity of this support and its role in making possible for mothers to be in the labour market varies considerably, depending on welfare state arrangements and the availability of part-time jobs (Albertini, 2016; Bordone et al., 2017; Di Gessa et al., 2016).

In the analysis of the growing role of grandparents in European societies the case of Italy is of particular interest. As a matter of fact, the country combines an interesting set of institutional, social and demographic characteristics that makes the social role of grandparents even more pivotal than elsewhere. In particular, the Italian welfare system is characterized by a scarce provision of childcare services for children between 0 and 3 years, a reduced level of child-related allowances and income transfers; moreover, the availability of part-time jobs on the labour market is relatively low. All of these characteristics make more difficult for parents to reconciliate work and child-related time demands, increasing the need of grandparental support. At the same time, the fact that Italian grandparents tend to retire from the formal labour market at lower ages than their European peers is likely to contribute to increasing the number of grandparents who are available to provide intensive support to their adult children (Naldini & Saraceno, 2008; Saraceno, 2018). These and other factors, specifically associated with the Italian family system, contribute to increasing the relevance of grandparents in the upbringing of their small grandchildren vs. what happens in other European countries. The strong ties connecting Italian grandparents and their grandchildren has not shown any sign of weakening in the last two decades, despite the changing labour market participation of grandmothers may challenge this institutional equilibrium in the next future (Arpino et al., 2012; Bratti et al., 2018; Del Boca et al., 2005; Pelle et al., 2021; Saraceno, 2018; Zanasi et al., 2022). Furthermore, it has been shown that if, on the one hand, intensive care provision to grandchildren is particularly common between married, female grandparents and among those who are in good health and have adequate economic resources; at the same time, in its less intensive forms grandparental support is a ubiquitous social phenomenon in Italy (Zamberletti et al., 2018).

When looking at the outcomes of grandparenting in Italy, similarly to what has been done in other countries, scholars have focused on several outcomes on couples and grandparents, including, e.g. mother’s participation in paid labour market and grandparents’ wellbeing. In particular, considering the effects on adult children, it has been shown that besides affecting children reproductive decisions (Aassve et al. 2012; Battistin et al., 2014; Bratti et al., 2018), grandparents’ support significantly and positively affects mother’s labour market attachment and grandchildren’s wellbeing (e.g. Ruiz & Silverstein, 2007). Adding to these studies, what we ask in the present study is if the effect of grandparents’ availability can also be observed at the level of conjugal life and, more specifically, on the extent to which housework tasks are equally shared between male and female partners who have minor children.

## The division of housework

There are different components of unpaid work related to family life. An important distinction is that between care work—i.e. looking after children, frail relatives and older family members—and other instrumental tasks such as household chores, paper work and family related consumption; within this latter type a further distinction proposed in the literature is that between routine tasks, typically performed by women, and intermittent tasks (e.g. household repairs, car maintenance) usually associated with men’s work (Coltrane, 2000; Lanchance-Grzela & Bouchard, 2010). Routine instrumental housework—i.e. cooking, cleaning, ironing, washing and shopping—is of particular interest: the inequality of its distribution between female and male partners appears to be particularly resistant both to long-term social changes and short-term shocks. Although gender equality has increased over the last decades and a “new” idea of father is emerging in many Western societies, roles concerning domestic work remain highly unequal (Grunow & Evetsson, 2019; Mencarini & Sironi, 2012; Zanatta, 1999). As a matter of fact, it has been observed that despite the growing share of women participating into paid labour market, the large part of these tasks still falls on their shoulders also when couples are highly educated and when men are unemployed (Carriero & Todesco, 2018). Differently, in recent decades the division of childcare work has become more equally distributed, and more so in highly educated egalitarian couples (Arrighi & Maume, 2000; Bianchi et al. 2012; Coltrane, 2000; Gershuny et al., 2005). The stickiness of the gendered division of routine housework has been further confirmed in the occurrence of a significant exogenous shock such as the recent pandemic—which has affected the organization of both working and family life. Zamberlan et al. (2021) observed that, in the UK, during the lock downs connected with the SARS-CoV-2 pandemic, male breadwinners whose paid hours reduced did not increase the share of time they invest in household chores, while women increased it disproportionately. Hank and Steinbach (2021), considering the German case, documented a shift towards the extremes of the distribution of housework during the pandemic—with men rarely taking on more than half of these duties.

Most of previous research on the micro-level determinants of the gendered division of household labour has focused on the role of time (time availability perspective), money and other economic resources (relative resources and economic dependency perspectives) and gender attitudes and ideology (doing gender perspective) (Aassve et al., 2014; Esping-Andersen et al. 2013; Horne et al., 2018; Lanchance-Grzela and Bouchard 2010). When considering meso- or macro-level factors, previous studies have taken into consideration the role of family–work reconciliation policies, maternity and parental leaves, the availability and cost of child care services, the prevalence at the national level of attitudes in favour of gender equality, and the prevalence of female employment (Fuwa & Cohen, 2007; Geist & Cohen, 2011; Knudsen & Wærness, 2008; Mandel et al., 2020).

From the point of view of the gendered division of housework Italy represents an interesting case. Long-term changes in the relative contribution of men to unpaid family work have been less marked here than in other European countries, and a traditional division remains prevalent: according to Carriero and Todesco (2018), in 2009 only 14% of partnered men shouldered 45% of more of domestic labour and only 7% of Italian couples equally shared routine tasks (see also Barigozzi et al., 2020; Carriero, 2009; Dotti Sani, 2012; Menniti et al., 2015; Zanella & De Rose, 2019). Furthermore, Dotti Sani (2016) documented that a gendered division in participation to domestic tasks is already present at very young ages, with daughters being significantly more likely to take up domestic tasks than their brothers. At the same time, Treas (2011) documents that Italian women, much more frequently than their peers in other countries, report turning to kin (vs. their husbands) for help with housework. While in other countries the large majority of married women prefer to turn to husbands to ask for help, in Italy husbands and kin are preferred by the same percentage of respondents (i.e. 48%). Next, Menniti et al. (2015) show that when other members—besides the nuclear family—are present in the household, time spent on housework decreases for both male and female partners, but the gender imbalance in its distribution remains. These findings, together with the important role of grandparents underlined above, make Italy a particularly interesting case for investigating the association between grandparents’ availability and the division of housework.

To the best of our knowledge, not much attention has been devoted to the potential role of informal support from family or non-family members in affecting the distribution of household work between male and female partners (Coltrane, 2000). The role of informal support—from any source—can clearly alter the (implicit or explicit) negotiations and equilibria within conjugal couples.

Previous studies adopting the time availability perspective have mostly focused on the bargaining process taking place between male and female partners (e.g. Gonalons-Pons, 2015). These analyses have also explored the role of labour market and welfare institutions—and in particular of child care policies—in affecting the outcome of these negotiation processes, but not the role of grandparental support. In a context, like the Italian one, where grandparents have such a relevant role that their availability even shapes young couples’ reproductive decisions, we think it is important to ask if and how the availability of this support may alter the equilibria in the gender division of housework. Grandparental support allows parents to completely outsource to them family work, or at least to unburden the two partners in carrying out specific household tasks. In both cases, grandparental support reduces the total amount of housework to be divided and negotiated within the couple. This may also reduce the chances to divide housework unequally: couples who receive help from grandparents may have less need to bargain over the amount of time devoted to housework. Studies show that families outsourcing housework tasks save time spent on the routine and time-intensive tasks that are often done by women (Bianchi et al., 2000; Gupta, 2007). Thus, women may benefit proportionally more than men from outsourcing or receiving grandparental support, because grandparents tend to provide help in routine and instrumental tasks (*decreasing inequality expectation*).

However, grandparental support may reduce the total amount of work, but leave unaltered the share of housework that men and women do, in line with what has been found by Menniti et al. (2015). Outsourcing family work may reduce the burden of unpaid family work without distributing the remaining homework differently, for instance when partners rearrange their tasks division to comply with societal norms and gender expectations. Women may compensate for the reduced burden by taking on new tasks and continue to be in charge of a similar proportion of the housework (Gonalons-Pons, 2015) (*unaltered inequality expectation*).

Alternatively, frequent interaction with own parents and parents-in-law may even increase inequality in the division of homework, when strong family ties reflect a traditional view of conjugal life and division of housework. Parents with more traditional family norms may value both intergenerational relationships and a traditional division of housework tasks (Carriero & Todesco, 2018). For instance, following the dimension of lineage, paternal grandparents may favour (grand)mother–son contact and be more prone to provide types of support that alleviate their son from his household tasks, thus further amplifying gender disparities in the division of housework (*increasing inequality expectation*).

In light of these (and other) different possible micro-level mechanisms, rather than formulating specific hypotheses about the direction of the potential effect of grandparents’ availability on the symmetry of the division of housework, we formulate a more general research question: is the potential availability of grandparental support associated with more or less inequality in the gendered division of household labour in couples with minor children?

## Data and methods

### Sample

The data utilized in the analyses are from the 2016 ISTAT “Family and Social Subjects” survey (FSS—*Famiglie e Soggetti Sociali*). The survey collects information on several aspects of family life, including family relationships, living arrangements and support exchange, every 5 years from 1998. In 2016 it reached about 24,700 individuals aged 18 or more, identified as heads of their households, residing in 850 different Italian municipalities; the survey response rate was equal to 78.2%.^1^ The analytical sample utilized in our empirical analyses is made of respondents who have at least one child aged below 18 years and living in the same household, and at least one living parent or parent in-law. Un-partnered individuals are excluded because our main variable of interest is the division of housework within couples. Respondents who only have children older than 18 years have been excluded on the basis of the assumption that grandparental support is less important when children are adult. We also exclude respondents who have missing information in one of the relevant indicators used in the analysis. The final sample includes 4991 respondents.

### Dependent variables

We use two dependent variables based on eight tasks performed within the household: shopping; cooking; cleaning; washing; ironing; paying bills, fixing up, and organizing social activities. The first dependent variable, i.e. division of housework within conjugal couples, is measured by an additive scale that takes into consideration all of the eight items. The second variable, which is an additive scale of the five items regarding “female type” of housework (shopping; cooking; cleaning; washing; and ironing), captures routine and instrumental activities that are done on a daily (or almost daily) basis. This distinction is consistent with previous studies showing that these are five of the most relevant routine tasks generally associated with “female-type” housework, whereas other non-routine tasks—such as doing small repairs—are generally associated with men’s duties. It is also worth noting that existing research has also reported that it is the distribution of “female type” housework that is more resistant to changes in its gendered distribution, even when new, less traditional models of partnerships become more common (Coltrane, 2000; Lanchance-Grzela and Bouchard 2010).

Respondents reported whether each of these five activities were done by: (1) only the respondent; (2) the respondent most of the time; (3) shared by both partners; (4) the partner most of the time; (5) only the partner, or (6) outsourced. The responses were utilized to build an additive index by adopting the following codification: “shared by the partners” and “outsourced” were both considered as the highest level of equal sharing of routine housework tasks, and given a score of 3 points; on the opposite, the categories “only the respondent” and “only the partner” were treated as the maximum level of inequality in the distribution of unpaid work, and given the score of 1; “respondent most of the time” and “the partner most of the time” were treated as an intermediate level, and given a score of 2. We chose to treat the categories “shared by partners” and “outsourced” together, because only few respondents report outsourcing domestic activities (see Appendix Table 4); moreover, outsourcing is often the result of a shared partners’ decision and, when services are bought on the market, shared economic resources.

**Fig. 1:**
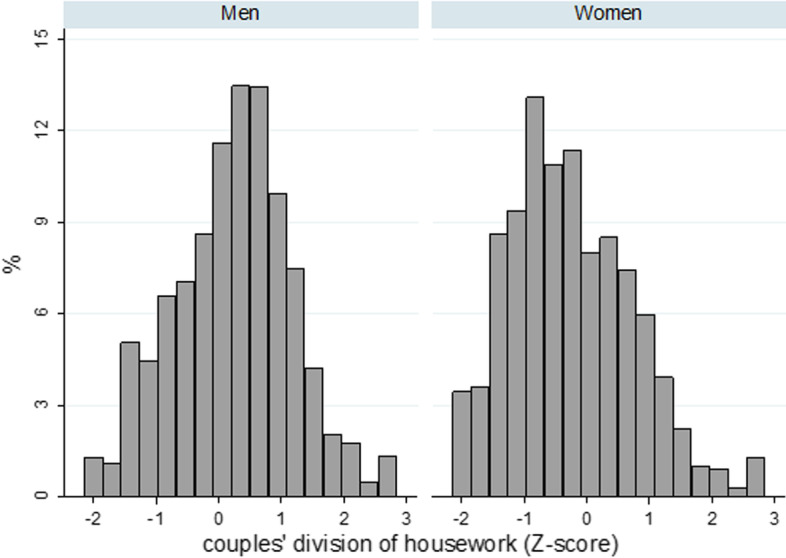
Symmetry in the division of household tasks according to respondent’s gender (8 items). Higher values, more symmetry

**Fig. 2:**
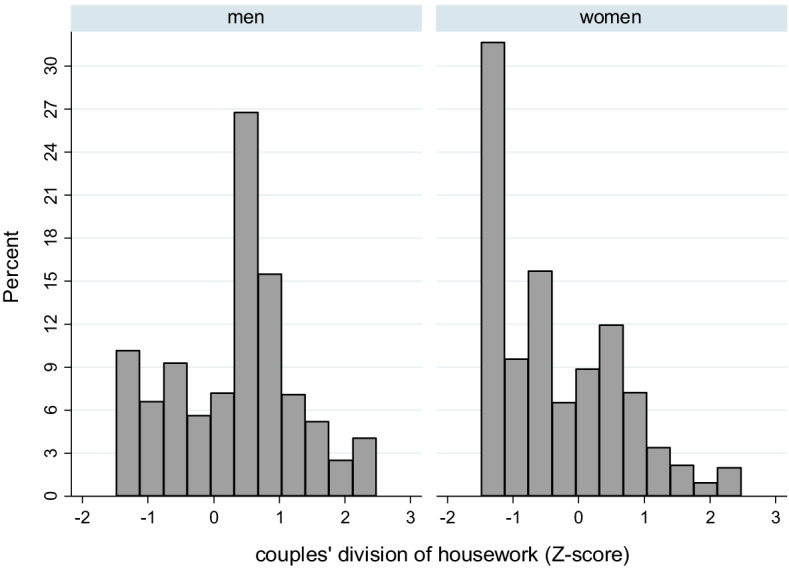
Symmetry in the division of instrumental household tasks according to respondent’s gender (5 items). Higher values, more symmetry

The sum of the scores of each individual’s answers to the eight items constitutes our measure of equality of the distribution of housework tasks, while the sum of the scores of the five items concerning routine activities is our measure of symmetry in the distribution of routine and instrumental tasks. Our dependent variables are therefore two indicators of equity in the division of housework, which captures more/less symmetry in couples’ division of unpaid labour. Notably, mothers reported less symmetry in couple’s division of housework than fathers do (see Figs. 1 and 2 in Appendix). There are opposite reasons that may lead to overreporting of own contribution to household tasks. On the one hand, mothers may want to display their central role in the family, while fathers may report a more equal distribution of housework as a socially desirable response in the framework of changing models of partnerships (Grunow & Evetsson, 2019). For this reason, the following analyses are performed for mothers and fathers separately. In multivariate analyses, we also use gender-specific standardized values of the dependent variable, i.e. we standardized the index according to its mean and standard deviation among men and women separately.

### Independent variables

In the 2016 wave of the FSS survey information on grandparental care received by respondents’ families was not collected. Nevertheless, in light of the results of many previous studies on the topic and on the Italian case, it is reasonable to utilize information on structural and associational solidarity (i.e. geographical distance and contact frequency) between adult children and older (grand)parents as a proxy of the potential availability of grandparents’ social support. Thus, our underlying assumption, which is supported by findings on Italian grandparents (Albertini & Tosi, 2018), is that face-to-face contact with grandparents provides an opportunity to receive support and outsource family work. Parent–adult child face-to-face contact frequency is used as a proxy of the potential and/or actual help that couples receive from grandparents and it represents our main independent variable.

Besides promoting solidarity, in-person interaction enables family members to exchange various forms of help and care (Rossi & Rossi, 1991). In previous research the frequency of face-to-face contact has been often considered as an indirect measure of intergenerational support, particularly in order to capture forms of instrumental support that are too idiosyncratic to measure in a questionnaire (Kalmjin & Dykstra, 2006). Since one respondent per household reported information about contact frequency with parents and parents-in-law, we used the most contacted grandparent to create an indicator of contact with grandparents. We also combined information on in-person contacts with that on intergenerational coresidence. As a matter of fact, as pointed out by previous studies (e.g. Glaser et al., 2018), intergenerational coresidence is a form of support that generations provide to each other—particularly in Southern European societies (Albertini & Kohli, 2013; Tosi, 2017). The answer categories were recoded into: (1) living in the same household; (2) daily contact; (3) weekly, which includes the categories “several meetings a week” and “once a week”; (3) less than weekly.

Independent control variables included in the analysis are the following: number of grandparents alive; number of children living at home; respondent’s age (25–34, 35–39, 40–44, 45–49, 50–59); macro geographical region of residence (north, centre, south and islands); respondent’s educational degree (lower than secondary, secondary, tertiary); respondent’s and partner’s employment status (in paid work, not in paid work), and receiving domestic help from a babysitter or housekeeper. Table 1 reports descriptive statistics for all the variables utilized in the analyses. Consistent with results from previous studies on the Italian labour market, mothers have on average a higher educational level but lower labour market participation than fathers. The proportion of respondents reporting to have bought support with housework or care on the market—i.e. housekeeper, or babysitter—is low. The average number of grandparents that the children of respondents have is high, also the frequency of face-to-face contacts with these is significant: more than 40% of respondents report seeing their parents/in-law on a daily basis.

**Table 1:** Sample descriptive statistics

	Men	Women
	% (*N*) or mean (SD)	% (*N*) or mean (SD)
Meetings with (grand)parents		
Daily	40.6 (1031)	46.3 (1148)
Living at home	4.1 (103)	4.6 (113)
Weekly	35.8 (910)	31.2 (774)
Less than weekly	19.5 (495)	17.9 (443)
N. of grandparents	3.1 (0.9)	3.1 (0.9)
Age		
25–34	12.2 (310)	20.3 (504)
35–39	16.2 (411)	21.1 (524)
40–44	23.4 (595)	26.4 (654)
45–49	25.5 (648)	21.5 (533)
50–59	22.6 (575)	10.6 (264)
Region		
North	45.1 (1145)	45.8 (1136)
Centre	16.7 (425)	17.2 (427)
South and islands	38.1 (969)	37.0 (918)
Educational level		
Tertiary	15.5 (394)	23.9 (595)
Secondary	48.6 (1233)	47.5 (1179)
Lower than secondary	35.9 (912)	28.5 (707)
Respondent is in paid work	91.8 (2331)	59.3 (1471)
Partner is in paid work	58.0 (1474)	91.2 (2264)
Hiring a housekeeper	4.8 (121)	6.4 (159)
Receiving support babysitter	2.7 (70)	3.7 (93)
Number of children living at home	1.8 (0.7)	1.8 (0.7)

## Results

Table 2 shows results from linear regression models on the gender symmetry in the division of housework tasks. As reported above, the relative majority of the respondents report having daily face-to-face contact with the most frequently met parent/parent in-law; with respect with this group all the other categories of our main independent variable are associated with a more symmetric division of routine housework tasks. Regression coefficients, tough, vary considerably in their size and statistical significance. When we focus our attention on those couples in which there is a lower than daily frequency of in-person meetings with the (grand)parents, we cannot safely argue that there is a clear association between associational family solidarity and a more (or less) unequal gendered division of housework. As a matter of fact, those who see members of the grandparents’ generation “weekly” and “less than weekly” tend to report a higher score in our symmetry index, but the differences vs. the reference group are barely significant, both from the substantive and statistical point of view. On the other hand, among couples with minor children who co-reside with at least one grandparent, we observe a statistically significant higher value of the symmetry index; in other words, the presence of a grandparent in the home is associated with a more gender-equal division of housework (*decreasing inequality expectation*). It is interesting to note that the size of the coefficient is similar, although slightly smaller, to that associated with the family hiring a housekeeper and it is markedly higher than that for having a babysitter. We formally compare the coefficients related to living with a grandparent, having a housekeeper and having a babysitter, by using Wald tests. The coefficient of coresidence is similar to that of having a housekeeper and larger than the one of receiving support from a babysitter. What is more, these results are consistent throughout the three different samples utilized in the analyses: overall, only male or female respondents.

**Table 2:** Linear regression models on symmetry in the division of housework (*Z*-score)

	Overall		Men		Women	
	Coef	S.E	Coef	S.E	Coef	S.E
Meetings with (grand)parents (Ref. Daily)						
Living at home	0.339**	(0.069)	0.328**	(0.097)	0.363**	(0.094)
Weekly	0.060^+^	(0.032)	0.059	(0.043)	0.055	(0.045)
Less than weekly	0.057	(0.039)	− 0.030	(0.053)	0.139*	(0.055)
N. of grandparents	0.034*	(0.016)	0.015	(0.022)	0.021	(0.022)
Age (Ref. 25–34)						
35–39	− 0.102*	(0.047)	− 0.188**	(0.071)	− 0.085	(0.060)
40–44	− 0.118**	(0.045)	− 0.176**	(0.067)	− 0.154**	(0.059)
45–49	− 0.103*	(0.047)	− 0.249**	(0.068)	− 0.107^+^	(0.064)
50–59	0.041	(0.054)	− 0.149*	(0.074)	− 0.045	(0.082)
Region (Ref. North)						
Centre	0.014	(0.039)	0.050	(0.053)	− 0.059	(0.054)
South	− 0.058^+^	(0.032)	− 0.074^+^	(0.044)	− 0.074	(0.045)
Education (Ref. Tertiary)						
Secondary	− 0.099**	(0.038)	− 0.096^+^	(0.056)	− 0.165**	(0.049)
Lower than secondary	− 0.138**	(0.042)	− 0.095	(0.059)	− 0.310**	(0.057)
Employed	0.381**	(0.058)	0.193^+^	(0.101)	0.304**	(0.076)
Working hours per week	0.002	(0.001)	− 0.007**	(0.002)	0.003	(0.002)
Employed partner	0.345**	(0.065)	0.036	(0.083)	0.332**	(0.117)
Partner working hours	− 0.010**	(0.002)	0.007**	(0.002)	− 0.010**	(0.002)
Hiring a housekeeper	0.352**	(0.063)	0.232*	(0.093)	0.439**	(0.083)
Babysitter	0.135^+^	(0.080)	0.237*	(0.117)	0.007	(0.105)
N. of children	− 0.053**	(0.018)	− 0.022	(0.026)	− 0.038	(0.026)
Constant	− 0.711**	(0.135)	− 0.088	(0.201)	− 0.637**	(0.182)
Observations	4991		2526		2465	
*R*-squared	0.085		0.053		0.109	

**p < 0.01, *p < 0.05, ^+^*p* < 0.1

Finally, it is worth noting that gender-specific mechanisms in the division of housework are related to respondents’ educational gradient and employment participation: women’s education and employment promote more symmetry in the division of housework tasks, while there is no statistically significant association between men’s education, employment and the division of household labour.

In Table 3, we focus on the symmetry index derived from routine instrumental activities (5 items). The results show that intergenerational coresidence between grandparents and grandchildren is associated with a more equal division of routine housework tasks. The coefficients are similar to those reported in Table 2, suggesting that living with grandparents helps couples to allocate time-consuming tasks more equally, too. Among women but not among men, the coefficient of coresidence is similar to that of having a housekeeper. This suggests that instrumental support in housework tasks helps couples to have a more equal division of housework (*decreasing inequality expectation*). On the contrary, we find no substantial associations between contact frequency and the symmetry index (*unaltered inequality expectation*). In the model on both men and women, having weekly meetings with grandparents is related to a higher score in the symmetry index, but the coefficient (0.07) is very small compared to the one of coresidence (0.3).

**Table 3:** Linear regression models on symmetry in the division of routine and instrumental housework tasks (5 items) (*Z*-score)

	Overall		Men		Women	
	Coef	S.E	Coef	S.E	Coef	S.E
Meetings with (grand)parents (Ref. Daily)						
Living at home	0.300**	(0.068)	0.301**	(0.096)	0.311**	(0.088)
Weekly	0.068*	(0.031)	0.064	(0.042)	0.061	(0.042)
Less than weekly	0.069	(0.040)	0.021	(0.052)	0.101^+^	(0.052)
N. of grandparents	0.030^+^	(0.016)	0.005	(0.022)	0.015	(0.021)
Age (Ref. 25–34)						
35–39	− 0.097*	(0.046)	− 0.174*	(0.070)	− 0.098^+^	(0.056)
40–44	− 0.103*	(0.044)	− 0.174**	(0.066)	− 0.149**	(0.055)
45–49	− 0.066	(0.046)	− 0.248**	(0.067)	− 0.069	(0.060)
50–59	0.064	(0.053)	− 0.175*	(0.073)	− 0.034	(0.077)
Region (Ref. North)						
Centre	0.023	(0.038)	0.052	(0.053)	− 0.042	(0.051)
South	− 0.091**	(0.031)	− 0.113**	(0.043)	− 0.104*	(0.042)
Education (Ref. Tertiary)						
Secondary	− 0.079*	(0.037)	− 0.049	(0.055)	− 0.186**	(0.046)
Lower than secondary	− 0.121**	(0.041)	− 0.059	(0.059)	− 0.341**	(0.054)
Employed	0.330**	(0.057)	0.135	(0.100)	0.255**	(0.071)
Working hours per week	0.005**	(0.001)	− 0.005**	(0.002)	0.005*	(0.002)
Employed partner	0.425**	(0.064)	0.096	(0.083)	0.361**	(0.110)
Partner working hours	− 0.013**	(0.001)	0.005*	(0.002)	− 0.012**	(0.002)
Hiring a housekeeper	0.378**	(0.062)	0.289**	(0.092)	0.447**	(0.078)
Babysitter	0.169*	(0.078)	0.227*	(0.115)	0.078	(0.099)
N. of children	− 0.055**	(0.018)	− 0.029	(0.025)	− 0.028	(0.024)
Constant	− 0.789**	(0.132)	− 0.040	(0.199)	− 0.758**	(0.171)
Observations	4991		2526		2465	
*R*-squared	0.119		0.054		0.134	

**p < 0.01, *p < 0.05, ^+^*p* < 0.1

### Robustness checks

We performed a number of robustness checks to test the sensitivity of our results to specific methodological choices. Three of these checks are worth reporting, and concern sample selection criteria and the specification of our regression model. First, we replicate our results on a subsample of couples with at least one child aged 13 or less living in the household (vs. couples with at least one minor children in the analyses reported above). It is common assumption in the literature, in fact, to consider children below age 14 as in need of a significant higher amount of care than older children. Thus, the potential support from grandparents may have a different effect on the division of housework within these families. The results of these additional analyses show that also in this subsample intergenerational coresidence between three generations is associated with more gender equity in the division of domestic labour. The symmetry in the gender division of routine housework tasks seems to be also associated with face-to-face contact frequency, with a slightly more equal division of housework among parents having less than daily contact.

A further check we performed regards the specification of the regression model: we include a control variable recording if the respondent has reported that at least one of the household activities has been outsourced, while still controlling for the hiring of housekeeper and/or a babysitter. The coefficients related to coresidence are smaller, but still significant when accounting for outsourcing at least one task (see Tables 5 and 6 in Appendix). Outsourcing therefore explains a marginal part of the association between coresidence with grandparents and couples’ division of domestic labour. The remaining association thus is likely to be due to activities that are not entirely outsourced to grandparents, but are unburdened by grandparental support.

Third, we add a control variable regarding traditional family norms. Traditional family norms and values may be correlated with both frequent interactions with grandparents and a higher asymmetry in the gendered division of routine housework. Traditional family norms are measured through an additive scale calculated on the basis of individuals’ responses to eight items (alpha equal to 0.72) about the following topics: cohabitation outside marriage; civil rights for homosexual couples; single mothers; children as the only source of realization for women; children as the only source of realization for men; divorce among unhappy couples; the priority of parents’ love for children’s development over their type of union (heterosexual or homosexual); the importance of living with the mother for children of divorced parents. The results of this additional analysis show that, indeed, individuals holding more traditional norms and values report a less balanced division of housework tasks. However, at the same time, the relation between our main independent variable and the symmetry index does not change. Thus, it seems that these norms do not explain the reported association between couples living with grandparents and a more equal division of housework (see Appendix, Tables 7 and 8).

## Discussion

Previous studies have shown the positive effect of grandparenting support on Italian mother’s labour market attachment and couples’ reproductive decisions. Far less is known about the role of grandparents in promoting equality in the gendered division of housework tasks. We suggested that while outsourcing family work to grandparents could help couples with minor children to have a more symmetric distribution of household labour (*decreasing inequality expectation*), frequent face-to-face contacts with grandparents could also be associated with more gender inequality because of traditional family norms that are both signalled and reinforced by a more intense contact with the older family generation (*increasing inequality expectation*). The results provide no evidence supporting these contrasting hypotheses, possibly because of a sort of compensation between these two conflicting mechanisms. We do not find a systematic and monotonic association between contact frequency with grandparents and symmetry in the gendered division of routine housework tasks. Daily meetings, which could be an opportunity to receive help from grandparents, do not seem to alter the asymmetric distribution of housework tasks within a couple. While frequent meetings between generations can be regarded as a form of emotional connection and a “light” form of help, intergenerational coresidence could be a source of intensive support in routine household tasks, including cleaning, washing, ironing and cooking (as recently suggested by Glaser et al., 2018). We find that intergenerational coresidence is associated with more gender equality in the division of domestic labour, arguably because it is connected with (partly) unburdening the conjugal couple from time-consuming activities that are usually done by women (decreasing inequality expectation). In fact, the positive role of living with grandparents for couples’ symmetry in routine household tasks is somewhat comparable to the one of receiving paid support from a housekeeper, and much larger than that of hiring a babysitter.

Grandparents live longer periods of their lives in good health and are increasingly able to provide rather than receive support. Consistent with Glaser et al. (2018), our findings suggest that, when they co-reside with their children and grandchildren, grandparents are more likely to give help in both irregular and routine household tasks, than to receive support from their adult children. This support tends to promote equality in the gendered division of household tasks, while having daily contacts does not have the same effect.

This study has five main limitations. First, our dependent variable on the gender symmetry in the division of household tasks is derived from questions directly answered by respondents. Social desirability may induce men to report a more equal division of labour, while women may want to display their central role in the family. In our analysis, we assume that these potential sources of bias, which are well-known in the literature (Aassve et al., 2014), are not correlated with face-to-face contact frequency and living arrangements. Second, in the FSS data information are reported from the point of view of the head of the household, which makes impossible to compare partners’ views within the same families. Both the division of domestic labour and the frequency of meeting with grandparents may differ in the perceptions of the two partners. Third, the sample size is too small to capture potential mechanisms of why intergenerational coresidence is associated with a more symmetric division of housework tasks. Therefore, additional data and analyses are needed to move from documenting to explaining this association. Four, the used data do not include the number of minutes dedicated by partners on each activity, which would provide a refined description of the division of domestic labour in Italian households. Five, contact frequency is used as a proxy for emotional and practical support, but its meaning may change remarkably when grandparental support concentrates on caregiving rather than on housework tasks.

These limitations are largely offset by several strengths of this study. We show that in a familialistic context characterized by high levels of traditionalism and intergenerational coresidence, living with a grandparent tends to promote a more equal division of housework tasks. One could think that coresidence and gender asymmetry in household tasks reflect a culture of strong family ties and traditional norms. On the contrary, our findings show that three-generation households have more equality in their division of both overall and routine housework tasks, given the support that grandparents provide.

## Data Availability

Information on how to obtain FSS data from ISTAT can be found here: https://www.istat.it/it/archivio/236637

## Appendix

See Table 4; Figs. 1, 2; Tables 5, 6, 7, 8, 9 and 10.

**Table 4:** Descriptive statistics of housework tasks

	Shopping	Cooking	Cleaning	Washing	Ironing	Fixing-up	Bills	Social
Men								
Only respondent	8.4	7.7	6.8	8.2	8.5	51.3	28.2	5.8
Respondent most	11.9	12.2	9.7	10.0	10.0	24.4	17.2	8.4
Shared by partners	37.0	20.0	19.5	10.7	7.2	8.1	26.7	65.2
Partner most	33.5	40.6	40.2	39.6	37.8	6.1	17.2	14.1
Only partner	8.9	18.2	21.8	30.5	32.0	3.1	9.0	5.2
Outsourced	0.2	1.2	2.0	0.9	4.5	7.0	1.7	1.7
Women								
Only respondent	40.1	57.0	62.1	73.0	72.5	6.1	25.0	12.2
Respondent most	24.1	23.9	22.7	17.7	15.1	5.9	9.7	8.6
Shared by partner	27.8	13.7	10.9	7.0	4.3	11.8	25.7	65.7
Partner most	6.5	3.6	0.9	1.0	1.0	40.8	24.1	8.8
Only partner	1.1	0.5	0.2	0.3	0.4	27.6	13.7	3.0
Outsourced	0.3	1.3	3.1	1.0	6.6	7.8	1.8	1.7

**Table 5:** Linear regression models on symmetry in the division of housework tasks (*Z*-score)

	Overall		Men		Women	
	Coef	S.E	Coef	S.E	Coef	S.E
Meetings with (grand)parents (Ref. Daily)						
Living at home	0.262**	(0.069)	0.267**	(0.096)	0.276**	(0.093)
Weekly	0.071*	(0.031)	0.071^+^	(0.042)	0.065	(0.044)
Less than weekly	0.071^+^	(0.039)	− 0.016	(0.053)	0.153**	(0.055)
N. of grandparents	0.035*	(0.016)	0.014	(0.022)	0.025	(0.022)
Age (Ref. 25–34)						
35–39	− 0.084^+^	(0.046)	− 0.163*	(0.070)	− 0.075	(0.059)
40–44	− 0.107*	(0.044)	− 0.172**	(0.066)	− 0.133*	(0.058)
45–49	− 0.089^+^	(0.047)	− 0.237**	(0.068)	− 0.089	(0.063)
50–59	0.048	(0.053)	− 0.142^+^	(0.073)	− 0.039	(0.081)
Region (Ref. North)						
Centre	0.017	(0.038)	0.038	(0.053)	− 0.038	(0.054)
South	− 0.060^+^	(0.032)	− 0.083^+^	(0.043)	− 0.065	(0.044)
Education (Ref. Tertiary)						
Secondary	− 0.068^+^	(0.037)	− 0.074	(0.055)	− 0.126**	(0.049)
Lower than secondary	− 0.106*	(0.041)	− 0.069	(0.059)	− 0.278**	(0.057)
Employed	0.384**	(0.058)	0.214*	(0.100)	0.312**	(0.075)
Working hours per week	0.001	(0.001)	− 0.007**	(0.002)	0.002	(0.002)
Employed partner	0.362**	(0.064)	0.056	(0.083)	0.340**	(0.116)
Partner working hours	− 0.010**	(0.001)	0.006**	(0.002)	− 0.010**	(0.002)
Hiring a housekeeper	0.205**	(0.064)	0.117	(0.094)	0.275**	(0.084)
Babysitter	0.098	(0.079)	0.210^+^	(0.116)	− 0.034	(0.104)
N. of children	− 0.054**	(0.018)	− 0.025	(0.025)	− 0.037	(0.025)
Outsourcing at least one activity	0.412**	(0.039)	0.350**	(0.055)	0.438**	(0.054)
Constant	− 0.608**	(0.133)	− 0.015	(0.200)	− 0.542**	(0.180)
Observations	4991		2526		2465	
*R*-squared	0.104		0.068		0.133	

***p* < 0.01, **p* < 0.05, ^+^*p* < 0.1

**Table 6:** Linear regression models on symmetry in the division of routine and instrumental housework tasks (5 items) (*Z*-score)

	Overall		Men		Women	
	Coef	S.E	Coef	S.E	Coef	S.E
Meetings with (grand)parents (Ref. Daily)						
Living at home	0.257**	(0.068)	0.276**	(0.096)	0.255**	(0.088)
Weekly	0.074*	(0.031)	0.069	(0.042)	0.067	(0.042)
Less than weekly	0.077*	(0.038)	0.026	(0.052)	0.110*	(0.052)
N. of grandparents	0.031^+^	(0.016)	0.004	(0.022)	0.018	(0.021)
Age (Ref. 25–34)						
35–39	− 0.087^+^	(0.046)	− 0.164*	(0.070)	− 0.092	(0.056)
40–44	− 0.096*	(0.044)	− 0.173**	(0.066)	− 0.135*	(0.055)
45–49	− 0.058	(0.046)	− 0.243**	(0.067)	− 0.057	(0.060)
50–59	0.068	(0.053)	− 0.172*	(0.073)	− 0.031	(0.076)
Region (Ref. North)						
Centre	0.024	(0.038)	0.047	(0.053)	− 0.028	(0.051)
South	− 0.093**	(0.031)	− 0.117**	(0.043)	− 0.098*	(0.042)
Education (Ref. Tertiary)						
Secondary	− 0.061^+^	(0.037)	− 0.041	(0.055)	− 0.161**	(0.046)
Lower than secondary	− 0.103*	(0.041)	− 0.049	(0.059)	− 0.321**	(0.054)
Employed	0.332**	(0.057)	0.143	(0.100)	0.260**	(0.071)
Working hours per week	0.005**	(0.001)	− 0.005**	(0.002)	0.004*	(0.002)
Employed partner	0.434**	(0.064)	0.104	(0.083)	0.367**	(0.109)
Partner working hours	− 0.014**	(0.001)	0.005*	(0.002)	− 0.012**	(0.002)
Hiring a housekeeper	0.295**	(0.063)	0.243**	(0.094)	0.341**	(0.080)
Babysitter	0.148^+^	(0.078)	0.216^+^	(0.115)	0.052	(0.098)
N. of children	− 0.055**	(0.018)	− 0.030	(0.025)	− 0.027	(0.024)
Outsourcing at least one activity	0.231**	(0.039)	0.141*	(0.055)	0.285**	(0.051)
Constant	− 0.731**	(0.132)	− 0.010	(0.200)	− 0.697**	(0.171)
Observations	4991		2526		2465	
*R*-squared	0.125		0.056		0.145	

***p* < 0.01, **p* < 0.05, ^+^*p* < 0.1

**Table 7:** Linear regression models on symmetry in the division of housework tasks (*Z*-score)

	Overall		Men		Women	
	Coef	S.E	Coef	S.E	Coef	S.E
Meetings with (grand)parents (Ref. Daily)						
Living at home	0.342**	(0.071)	0.326**	(0.099)	0.364**	(0.096)
Weekly	0.066*	(0.032)	0.072^+^	(0.043)	0.056	(0.045)
Less than weekly	0.087*	(0.040)	0.002	(0.054)	0.173**	(0.057)
N. of grandparents	0.036*	(0.016)	0.017	(0.023)	0.025	(0.023)
Age (Ref. 25–34)						
35–39	− 0.121*	(0.047)	− 0.202**	(0.072)	− 0.110^+^	(0.060)
40–44	− 0.120**	(0.045)	− 0.171*	(0.068)	− 0.161**	(0.059)
45–49	− 0.116*	(0.048)	− 0.260**	(0.069)	− 0.121^+^	(0.065)
50–59	0.016	(0.055)	− 0.154*	(0.075)	− 0.098	(0.082)
Region (Ref. North)						
Centre	0.024	(0.039)	0.057	(0.054)	− 0.053	(0.055)
South	− 0.046	(0.033)	− 0.056	(0.044)	− 0.062	(0.046)
Education (Ref. Tertiary)						
Secondary	− 0.087*	(0.038)	− 0.073	(0.056)	− 0.155**	(0.049)
Lower than secondary	− 0.112**	(0.042)	− 0.056	(0.060)	− 0.286**	(0.058)
Employed	0.361**	(0.059)	0.143	(0.103)	0.292**	(0.077)
Working hours per week	0.002	(0.001)	− 0.006**	(0.002)	0.003	(0.002)
Employed partner	0.341**	(0.066)	0.048	(0.084)	0.285*	(0.118)
Partner working hours	− 0.010**	(0.002)	0.006**	(0.002)	− 0.010**	(0.002)
Hiring a housekeeper	0.346**	(0.064)	0.223*	(0.094)	0.438**	(0.084)
Babysitter	0.148^+^	(0.080)	0.243*	(0.117)	0.011	(0.106)
N. of children	− 0.049*	(0.019)	− 0.016	(0.026)	− 0.031	(0.026)
Traditional family norms	− 0.010**	(0.003)	− 0.011*	(0.004)	− 0.014**	(0.004)
Constant	− 0.528**	(0.151)	0.123	(0.225)	− 0.332	(0.204)
Observations	4835		2449		2386	
*R*-squared	0.086		0.054		0.113	

***p* < 0.01, **p* < 0.05, ^+^*p* < 0.1

**Table 8:** Linear regression models on symmetry in the division of routine and instrumental housework tasks (5 items) (*Z*-score)

	Overall		Men		Women	
	Coef	S.E	Coef	S.E	Coef	S.E
Meetings with (grand)parents (Ref. Daily)						
Living at home	0.298**	(0.069)	0.279**	(0.097)	0.318**	(0.090)
Weekly	0.072*	(0.031)	0.072^+^	(0.043)	0.062	(0.042)
Less than weekly	0.102**	(0.039)	0.054	(0.053)	0.137**	(0.053)
N. of grandparents	0.033*	(0.016)	0.010	(0.022)	0.018	(0.021)
Age (Ref. 25–34)						
35–39	− 0.112*	(0.046)	− 0.193**	(0.071)	− 0.115*	(0.057)
40–44	− 0.097*	(0.044)	− 0.167*	(0.067)	− 0.146**	(0.056)
45–49	− 0.079^+^	(0.047)	− 0.256**	(0.068)	− 0.089	(0.061)
50–59	0.044	(0.053)	− 0.168*	(0.074)	− 0.088	(0.077)
Region (Ref. North)						
Centre	0.029	(0.038)	0.062	(0.053)	− 0.043	(0.052)
South	− 0.080*	(0.032)	− 0.089*	(0.044)	− 0.097*	(0.043)
Education (Ref. Tertiary)						
Secondary	− 0.064^+^	(0.037)	− 0.022	(0.056)	− 0.175**	(0.047)
Lower than secondary	− 0.095*	(0.041)	− 0.017	(0.060)	− 0.317**	(0.055)
Employed	0.313**	(0.058)	0.082	(0.101)	0.247**	(0.072)
Working hours per week	0.005**	(0.001)	− 0.005*	(0.002)	0.005*	(0.002)
Employed partner	0.425**	(0.064)	0.108	(0.083)	0.324**	(0.111)
Partner working hours	− 0.014**	(0.001)	0.004^+^	(0.002)	− 0.011**	(0.002)
Hiring a housekeeper	0.374**	(0.063)	0.290**	(0.093)	0.443**	(0.079)
Babysitter	0.178*	(0.079)	0.232*	(0.115)	0.074	(0.100)
N. of children	− 0.050**	(0.019)	− 0.025	(0.026)	− 0.017	(0.025)
Traditional family norms	− 0.010**	(0.003)	− 0.012**	(0.004)	− 0.016**	(0.004)
Constant	− 0.596**	(0.148)	0.184	(0.222)	− 0.413*	(0.192)
Observations	4858		2461		2397	
R-squared	0.120		0.056		0.137	

***p* < 0.01, **p* < 0.05, ^+^*p* < 0.1

**Table 9:** Linear regression models on symmetry in the division of housework tasks (*Z*-score). Sample of grandchildren aged 0–13

	Overall		Men		Women	
	Coef	S.E	Coef	S.E	Coef	S.E
Meetings with (grand)parents (Ref. Daily)						
Living at home	0.371**	(0.074)	0.324**	(0.104)	0.422**	(0.100)
Weekly	0.099**	(0.035)	0.118*	(0.047)	0.075	(0.049)
Less than weekly	0.082^+^	(0.043)	0.006	(0.059)	0.160**	(0.062)
N. of grandparents	0.022	(0.018)	− 0.000	(0.026)	0.009	(0.025)
Age (Ref. 25–34)						
35–39	− 0.094*	(0.047)	− 0.190**	(0.071)	− 0.072	(0.061)
40–44	− 0.116*	(0.046)	− 0.185**	(0.067)	− 0.161**	(0.061)
45–49	− 0.103*	(0.051)	− 0.261**	(0.072)	− 0.112	(0.072)
50–59	0.024	(0.065)	− 0.216*	(0.084)	− 0.011	(0.111)
Region (Ref. North)						
Centre	0.012	(0.043)	0.051	(0.058)	− 0.065	(0.060)
South	− 0.041	(0.035)	− 0.046	(0.048)	− 0.051	(0.050)
Education (Ref. Tertiary)						
Secondary	− 0.125**	(0.040)	− 0.132*	(0.060)	− 0.173**	(0.053)
Lower than secondary	− 0.146**	(0.045)	− 0.126*	(0.064)	− 0.302**	(0.063)
Employed	0.359**	(0.064)	0.133	(0.112)	0.287**	(0.084)
Working hours per week	0.002	(0.001)	− 0.006**	(0.002)	0.003	(0.002)
Employed partner	0.377**	(0.072)	0.103	(0.094)	0.271*	(0.129)
Partner working hours	− 0.011**	(0.002)	0.005*	(0.003)	− 0.010**	(0.003)
Hiring a housekeeper	0.375**	(0.070)	0.227*	(0.102)	0.472**	(0.091)
Baby-sitter	0.123	(0.080)	0.233*	(0.117)	0.002	(0.107)
N. of children	− 0.069**	(0.020)	− 0.036	(0.028)	− 0.051^+^	(0.028)
Constant	− 0.641**	(0.143)	0.027	(0.214)	− 0.557**	(0.195)
Observations	4103		2054		2049	
*R*-squared	0.086		0.057		0.108	

***p* < 0.01, **p* < 0.05, ^+^*p* < 0.1

**Table 10:** Linear regression models on symmetry in the division of routine and instrumental housework tasks (5 items) (*Z*-score)

	(1)	(2)	(3)	(4)	(5)	(6)
	Coef	se	Coef	se	Coef	se
Meetings with (grand)parents (Ref. Daily)						
Living at home	0.316**	(0.072)	0.293**	(0.103)	0.342**	(0.094)
Weekly	0.106**	(0.034)	0.109*	(0.046)	0.090^+^	(0.046)
Less than weekly	0.088*	(0.042)	0.046	(0.058)	0.118*	(0.058)
N. of grandparents	0.014	(0.018)	− 0.011	(0.025)	− 0.006	(0.024)
Age (Ref. 25–34)						
35–39	− 0.097*	(0.046)	− 0.185**	(0.070)	− 0.098^+^	(0.057)
40–44	− 0.096*	(0.045)	− 0.182**	(0.066)	− 0.156**	(0.057)
45–49	− 0.055	(0.050)	− 0.258**	(0.071)	− 0.058	(0.067)
50–59	0.063	(0.063)	− 0.240**	(0.083)	0.057	(0.104)
Region (Ref. North)						
Centre	0.009	(0.042)	0.033	(0.057)	− 0.049	(0.056)
South	− 0.073*	(0.035)	− 0.089^+^	(0.048)	− 0.073	(0.047)
Education (Ref. Tertiary)						
Secondary	− 0.103**	(0.039)	− 0.083	(0.059)	− 0.190**	(0.050)
Lower than secondary	− 0.114**	(0.044)	− 0.065	(0.063)	− 0.330**	(0.059)
Employed	0.349**	(0.062)	0.139	(0.111)	0.271**	(0.079)
Working hours per week	0.004**	(0.001)	− 0.005**	(0.002)	0.004*	(0.002)
Employed partner	0.446**	(0.071)	0.154^+^	(0.092)	0.306*	(0.122)
Partner working hours	− 0.014**	(0.002)	0.004	(0.002)	− 0.011**	(0.002)
Hiring a housekeeper	0.388**	(0.068)	0.292**	(0.101)	0.447**	(0.086)
Babysitter	0.152^+^	(0.079)	0.226^+^	(0.116)	0.066	(0.101)
N. of children	− 0.073**	(0.020)	− 0.046^+^	(0.027)	− 0.040	(0.027)
Constant	− 0.700**	(0.140)	0.034	(0.212)	− 0.628**	(0.184)
Observations	4121		2064		2057	
*R*-squared	0.120		0.058		0.132	

Sample of grandchildren aged 0–13

***p* < 0.01, **p* < 0.05, ^+^*p* < 0.1

## Footnotes

1 The previous waves (1998; 2003; 2009) are not used in this study due to changes in the data collection over time. One of the most relevant differences is that FSS 2016 collects information on household heads only, whereas all household members were interviewed in previous waves.
